# Differential H3K9me2 heterochromatin levels and concordant mRNA expression in postmortem brain tissue of individuals with schizophrenia, bipolar, and controls

**DOI:** 10.3389/fpsyt.2022.1006109

**Published:** 2022-10-26

**Authors:** Hooriyah S. Rizavi, Kayla A. Chase, Chunyu Liu, Hannah Gavin, Cherise Rosen, Cuihua Xia, Alessandro Guidotti, Rajiv P. Sharma

**Affiliations:** ^1^Department of Psychiatry, The Psychiatric Institute, University of Illinois at Chicago, Chicago, IL, United States; ^2^Jesse Brown Veterans Affairs Medical Center, Chicago, IL, United States; ^3^Department of Biochemistry and Molecular Biology, University of Illinois at Chicago, Chicago, IL, United States; ^4^Department of Psychiatry, SUNY Upstate Medical University, Syracuse, NY, United States; ^5^School of Life Sciences, Central South University, Changsha, China

**Keywords:** schizophrenia, histone methylation, heterochromatin, postmortem brain, ChIP-Seq, gene expression

## Abstract

The existence of repressive and durable chromatin assemblies along gene promoters or networks, especially in the brain, is of theoretical and therapeutic relevance in a subset of individuals diagnosed with schizophrenia who experience a chronic, persistent, and treatment-resistant trajectory. We used chromatin immunoprecipitation followed by deep sequencing (ChIP-Seq) to generate an epigenomic map that includes differential sites occupied by di-methylated lysine 9 of histone 3 (H3K9me2), a repressive modification that is yet unexplored in human postmortem brain tissue. We have discovered over 150 significantly differential promoter sites in the postmortem prefrontal cortex tissue of individuals diagnosed with schizophrenia (*n* = 15) when compared to controls (*n* = 15). Potentially dysregulated gene categories include postsynaptic proteins, processing enzymes (for proproteins, lipids, and oxidative stress), cadherin family genes, the complement system, and peptide hormones. Ten genes with significantly increased or decreased H3K9me2 promoter occupation were selected through statistical analysis, function, or previous GWAS association, and Quantitative RT-PCR (qRT-PCR) was performed on an extended sample of postmortem brain tissue, adding an additional 17 controls, 7 individuals with schizophrenia, and 19 individuals with bipolar samples (*n* = 32 control, 22 schizophrenia, 19 bipolar). This approach revealed that mRNA expression levels correlated with chromatin modification levels in eight of 10 selected genes, and mRNA expression in the total sample could be predicted by the occupancy of H3K9me2. Utilization of this method and replication in a larger sample open a pathway to durable and restrictive epigenomic assemblies whose accumulation across the lifespan of individuals diagnosed with schizophrenia may explain treatment resistance, and advance therapeutic options.

## Introduction

The molecular basis of chronic, persistent, and treatment resistance in a subset of individuals diagnosed with schizophrenia (SCZ) can be informed by the study of durable tertiary cellular structures. Heterochromatin is one such structure and is seeded, assembled, and distributed along the chromosome by the post-translational modification of multiprotein components, as well as the underlying DNA strand. Because SCZ is characterized by long-term dysfunction in the frontal cortex, it follows that altered gene expression may be coordinated with the local epigenetic architecture of particular promoters and other regulatory sequences. Conceptually, studying these types of durable epigenetic modifications makes possible a dissection of a spatial/temporal stream of pathological events (immune, metabolic, psychological, stress, pharmacological) deposited in this molecular repository.

Heterochromatin that is characterized by the methylation of lysine 9 along the H3 histone tail (H3K9me2) is a chromatin assembly that is particularly linked to transcriptional repression. This type of chromatin assembly is often called “facultative heterochromatin” but is best described as permissively restrictive. H3K9me2 heterochromatin is located in gene-rich stretches of the genome and can facilitate nuclear localization and compartmentalization (1). Functionally, this modification can be distinguished from H3K9me1 or H3K9me3 and is targeted by histone methyltransferases (HMTs) such as G9a, GLP, and SETDB1 (2). H3K9me2 serves as a “ligand” for ambient “receptor” proteins of the heterochromatin family, particularly heterochromatin protein 1γ (HP1γ), which can be dislodged from this attachment by phosphorylation of the adjacent serine (H3S10-phos). The entire assembly anchored at H3K9me2/3 can include an array of epigenetic repressors such as Histone deacetylases (HDACs), DNA methyltransferases (DNMTs), and anchor platform adaptor proteins such as REST/CoREST as well as several identified lncRNAs that can cobble together these multiple components. Recent findings have identified up to 172 proteins embedded in this H3K9me2/3 seeded assembly (3, 4). This multiprotein complex is typically coordinated with local CpG methylation, another potentially lifelong covalent modification (5), providing a durable anchor for these repressive complexes (6, 7). H3K9me2 heterochromatin is dynamic and can assemble in response to oxidative stress (neurons) or prolonged kinase signaling (e.g., endotoxin tolerance in macrophages) (8–10). Once nucleated, H3K9me2/3 heterochromatin can spread up to distances of 15 kb along the DNA strand (11) and can sequester large regions of the epigenome to the nuclear periphery rendering these regions resistant to gene activation (1, 12, 13).

The ENCODE project has H3K9me2 data on 14 immortalized cell lines and three *in vitro* differentiated cell types. The three differentiated cell types include one bipolar neuron, one neural cell, and one hepatocyte. The Roadmap Epigenomics and PsychENCODE projects have not yet generated data on this histone modification. In other words, H3K9me2 has not been studied in a human population sample, particularly in the brain. Given the deficiency of scientific literature in this area, there is urgency to develop a reference map of H3K9me2 in human brains and explore H3K9me2 in individuals who experience chronic, persistent, and treatment resistant psychosis and in non-clinical controls.

Our laboratory has previously reported elevated global levels of H3K9me2 protein in the parietal cortex from postmortem brain tissue samples of individuals diagnosed with SCZ (14). Additionally, we identified elevated mRNA levels of the enzymes responsible for catalysis of H3K9me2 (GLP, G9a, and SETDB1) in postmortem brain tissue, as well as peripheral blood mononuclear cells (PBMCs) from individuals with SCZ (14, 15). Finally, we reported that this assembly is reversible by small molecule inhibition of its methyltransferases (16, 17) as well as kinase inhibitors modifying histone phosphorylation on site specific promoters (17). Because global protein levels do not identify the regulation of individual genes, we advanced these findings with a promoter-targeted approach. Here, we pioneered the examination of genome-wide occupancy of H3K9me2 in the postmortem frontal cortex tissue of individuals with schizophrenia and attempted to understand whether causation can be implied through the increase or decrease of gene expression in networks crucial to natural development and function.

Chromatin immunoprecipitation followed by deep sequencing (ChIP-Seq) for H3K9me2 in the postmortem brain tissue of individuals with schizophrenia has not previously been performed and can provide an illustrative example of the benefits of epigenome mapping. For logistical reasons, we have restricted the ChIP-Seq analysis to promoter regions in 30 prefrontal cortex samples [15 non-psychiatric controls (NPC) and 15 SCZ]. We selected H3K9me2 since it is a strongly repressive type of heterochromatin associated with euchromatin sectors of the genome, and conceptually could be reversed to allow facile expression of genes in the underlying and coordinate regions. In order to determine if the identified expression changes secondary to heterochromatin were specific to individuals with SCZ we directly measured mRNA levels using qRT-PCR in a larger and inclusive cohort of brain samples (NPC, *n* = 32, SCZ, *n* = 22, BPD, *n* = 19) that included individuals diagnosed with bipolar disorder (BPD). Finally, we performed a correlational analysis between mRNA expression and H3K9me2 promoter occupation in the brain.

## Materials and methods

### Postmortem brain tissue samples

Human postmortem brain tissue samples [Prefrontal cortex, Brodmann area 9 (PFC)] were obtained from the Harvard Brain Tissue Resource Center that were donated by individuals diagnosed with schizophrenia, bipolar, and controls. ChIP-Seq for the repressive modification H3K9me2 was performed on 30 postmortem PFC from individuals with SCZ (*n* = 15) and NPC (*n* = 15). ChIP-Seq mRNA validation using qRT-PCR in a larger cohort of brain samples from the same collection included three diagnostic groups NPC (*n* = 32), individuals with SCZ (*n* = 22), and individuals with BPD (*n* = 19). This expanded set included the 15 NPC and 15 individuals with SCZ samples that were initially analyzed with ChIP-seq. A summary of demographic parameters is given in Table 1.

**TABLE 1:** Subject demographics and clinical characteristics.

	Patient cohort		
	
	NPC (*n* = 32)	SCZ (*n* = 22)	BPD (*n* = 19)
M/F ratio	21/11	15/7	4/15
Age, years	59.6 ± 14.8	60.6 ± 12.5	62.8 ± 17.8
Postmortem interval, hours	21.8 ± 3.6	23.9 ± 7.3	22.2 ± 5.1
Brain pH	6.4 ± 0.3	6.5 ± 0.3	6.6 ± 0.3
Medication			
Antipsychotic drug use^a,b^	0	11	8

Values are expressed as mean ± SD.

^a^Present at the time of death.

^b^Includes the following with combination: clozapine, trifluoperazine, olanzapine, quetiapine, haloperidol, chlorpromazine, aripiprazole, thioridazine, perphenazine, risperidone, fluphenazine.

### Chromatin immunoprecipitation with sequencing

ChIP was performed according to a previously published procedure (16). Briefly, an aliquot of human brain tissue was used for each experiment and homogenized in 500 μl of RPMI1640 media (GIBCO #11875-093). Proteins were cross-linked to DNA by adding 48.25 μl of methanol-free formaldehyde (Thermo #28908) and incubated at 37°C for 5 min. The sample was then quenched with 70.5 μl of 1M glycine, spun down, the supernatant removed, and washed with PBS in the presence of 1:100 protease inhibitor (Calbiochem #539134). The samples were spun-down again, the supernatant removed, and resuspended in SDS lysis buffer (1% SDS, 10 mM EDTA, 50 mM Tris-HCl, pH 8.1), again in the presence of 1:100 protease inhibitor. Samples were then sonicated for 20 min at 10%df in a Covaris m220 sonicator. Further processing was performed using a ChIP assay kit (#17-295; Millipore, Upstate), and DNA was precipitated by standard ethanol precipitation. Lysates were immunoprecipitated (IP) with H3K9me2 monoclonal antibody (Abcam, ab1220) that was validated by both peptide ELISA and Western blot and is specific to only H3K9me2, not H3K9, H3K9me1, H3K9me3, H3K27me2/3, or H3K4me1/2/3, as verified in previous ChIP studies (17–19). Samples were then examined for quality prior to sequencing through qPCR for previously established negative (GAPDH) and positive (ZNF333 and UGT1A10) control genes for the H3K9me2 modification (20) (Supplementary Figure 1A).

### Quality control

Sample quality was examined in the following ways. First, a small quantity of the sonicated chromatin was run on an agarose gel to ensure fragment size was within 500–1,000 bp. Secondly, after IP and DNA extraction, DNA concentrations of the input and IP were measured *via* Qubit fluorometric assay. Finally, pulldown efficiency was determined by qPCR of the IP product. We verified GAPDH as a transcriptionally active promoter (not containing H3K9me2) and two genes ZNF333 and UGT1A10 as transcriptionally inactive promoters and thus would contain high H3K9me2 occupancy (Supplementary Figure 1) (20). There was no qPCR amplification of GAPDH promoter, while the ZNF333 and UGT1A10 show amplification and thus successful pulldown by the antibody. Samples that exhibited GAPDH promoter pulldown were discarded because of cross-reactivity or contamination and were not used for library preparation or sequencing (for example, sample #1113 in Supplementary Figure 1). These samples were reprocessed using fresh brain tissue for successful pulldown if necessary.

### Library construction and sequencing

After completion of the ChIP-Seq protocol, frozen immunoprecipitates were sent to the University of Illinois at Urbana Champaign for library construction and sequencing on an Illumina HiSeq 2500, followed by a data quality control pipeline. Samples were run at four samples per lane, returning 130–180 million reads, putting the average sequencing depth for each sample at approximately 40 million single-end reads (21, 22). We utilized single-end reads due to our focus on promoter regions, as the information on duplications, inversions, splice variants, or SNPs provided by double-end reads was not a priority. The quality of the runs was determined by FASTQC. An example of an acceptable FASTQC output from our data is provided in Supplementary Figure 1B.

### Alignment of sequenced reads

Reads were trimmed at both ends using Trimmomatic (23). Genome alignment was performed using Bowtie2, which was selected for its utility in aligning short DNA sequence reads to long mammalian genomes (24, 25). These alignments were visualized using the UCSC Genome Browser; examples for two selected genes are shown in Supplementary Figure 2. Multiple aligned sequences and duplicate reads were discarded before normalization. We limited our region of interest to a 10 kb window centered at the transcription start site (TSS)—a defined promoter region of fixed but approximate length since the upstream boundaries of most promoter regions are not well defined.

### GWAS enrichment analysis

Starting with the Working Group of Psychiatric Genomics Consortium 1 (PGC) SCZ GWAS summary statistics, we searched the significant differentially modified promoters from our data set against the 25,069 genes. Next we performed the hypergeometric distribution test using the software R 4.1.0 to see which promoters were enriched in the PGC3 SCZ GWAS significant associated genes. The PGC3 SCZ GWAS summary statistics (PGC3_SCZ_wave3.primary.autosome.public.v3.vcf.tsv.gz) was downloaded from PGC website,^1^ and the SNPs were annotated to genes using the software ANNOVAR (26) based on hg19_avsnp142 database (Supplementary Table 2).

### Identification and analysis of H3K9me2 sites in cases and controls

DiffReps were used to detect differential H3K9me2 modification sites, again limiting our search to the promoter regions of annotated genes (27). According to the evaluation of tools for ChIP-seq data analysis, this is the best choice for data analysis with biological replicates without predefined regions of interest (28). DiffReps analyzes an entire ChIP-Seq dataset using a fix-sized sliding genomic window, where only the reads falling inside the window are counted. The sliding window analyzes a fixed length as it is moved along the genome in fixed steps (e.g., window size 1 Kb moving along at 100 bp per step). The sliding window strategy is advantageous for a more “smeared” modification such as H3K9me2, since it is independent of any “peak” calling program. DiffReps output data includes not only nearby gene annotation but also other gene features (e.g., gene body, centromere, promoter, gene desert) allowing us to focus on gene promoters. DiffReps calculates case-control group difference directly, allowing us to identify gene-promoters based on fold change as well as both raw and Padj values (FDR) to determine a significant difference between cases and controls.

### Quantitative RT-PCR

Expanding the sample of postmortem brain tissue to include the same original ChIP-seq samples and an additional 17 NPC, 7 individuals with SCZ, and 19 individuals with BPD, total RNA was isolated using TRIzol reagent (Life Technologies) and treated with DNase (Ambion/Life Technologies #AM1906) after extraction. Total RNA was used to prepare cDNA *via* the Applied Biosystems High Capacity cDNA Reverse Transcription Kit (#4368814). For detection and measurement of expression, Fermentas Maxima SYBR Green/ROX qPCR Master Mix (#K0222) was used. PCR mixtures were run on a Thermo Scientific PikoReal real-time PCR System using manufacturer cycling conditions. Cycle threshold (CT) value was used for relative quantification, and all values were normalized to GAPDH, and run in duplicate. Ten genes were selected for qPCR assessment, taking into account combinatorial best fold change and adjusted *p*-values from our ChIP-seq data Table. Primer sequences are provided in Supplementary Table 3.

## Results

### Differential heterochromatin occupancy of 159 promoters

DiffReps output data contained 159 differentially modified promoters based on a raw *p*-value of *p* < 0.01, once all other gene features (intergenic areas, pericentromere, etc.) were cleared from the output (Supplementary Table 1). Of the 159 promoters, 82 have increased H3K9me2 occupancy, while 77 have decreased occupancy in individuals with SCZ when compared to NPC. Five genes (*AKT3, C4B, FURIN, GPX6, and NRGN*) were common to the PGC2 SCZ GWAS data set available to us during the project, and three of these genes (*AKT3, FURIN, GPX6*) remained common to the most recent PGC3 SCZ GWAS data set. Three genes, *FURIN, GPX6*, and *NRGN* have increased H3K9me2 promoter occupancy and two genes, *AKT3* and *C4B* show decreased promoter occupancy. Five additional genes (*GPHN, OXT, CDHR2, CDH20, LIPJ)* were selected, two (*GPHN, OXT)* based on previous associations in the SCZ literature (29, 30) and three (*CDHR2, CDH20, LIPJ*) are novel with no consistent link to individuals with SCZ. C4B was included with an additional focus of its identification with the PGC2 GWAS analysis (2014) and its central role in immune function; an isotypic allele C4A was noted to be significantly higher in the brain of individuals with SCZ (31). The statistical analysis of diffReps on the ten regions chosen to be analyzed using qPCR is reported in Table 2. The full list of 159 promoter regions with differentially modified H3K9me2 occupancy is provided in Supplementary Table 1.

**TABLE 2:** DiffReps output.

Gene	SCZ avg reads	NPC avg reads	Occupation	log2FC	*P*-value	Padj
		
	*n* = 15	*n* = 15				
**Increased H3K9me2 promoter occupation in SCZ compared to NPC**						
GPHN	140.72	54.22	Increased	1.38	2.89E-10	2.13E-07
CDHR2	61.97	10.07	Increased	2.62	1.13E-10	8.67E-08
GPX6	68.9	32.26	Increased	1.09	7.51E-07	9.54E-04
OXT	33.57	3.87	Increased	3.12	2.04E-07	9.23E-05
FURIN	169.84	166.46	Increased	0.03	1.08E-10	8.57E-07
NRGN	86.06	62.46	Increased	0.46	2.61E-04	9.12E-02
**Decreased H3K9me2 promoter occupation in SCZ compared to NPC**						
AKT3	26.74	515.22	Decreased	–4.27	1.38E-07	3.85E-04
CDH20	74.88	209.92	Decreased	–1.49	3.33E-16	3.65E-13
LIPJ	1.29	53.45	Decreased	–5.37	1.55E-15	1.62E-12
C4B	256.53	619.55	Decreased	–1.27	6.3E-03	2.9E-01

DiffReps provides a large amount of information about differentially modified genomic areas, which is reduced for relevance. “avg reads” is the average occupation of the gene promoter by H3K9me2 modification of all samples (SCZ; n = 15, NPC; n = 15). Log_2_FC refers to the log ratio of the fold change between NPC and SCZ average reads. P-values demonstrate high significance, while Padj value corresponds to p-value adjusted for multiple testing using Benjamini-Hochberg method. The complete DiffReps output is presented in Supplementary Table 1.

### mRNA expression is coordinated with H3K9me2 occupancy

**FIGURE 1:**
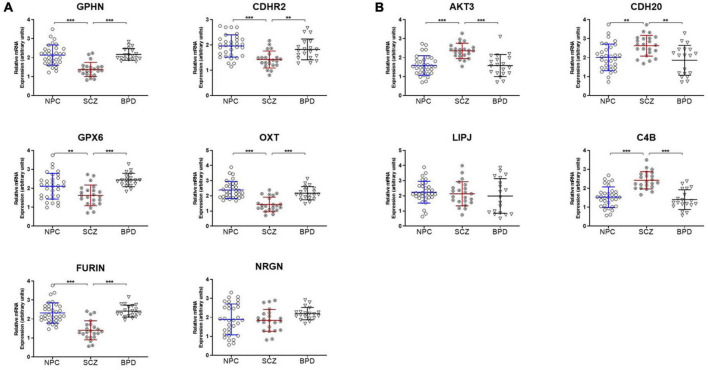
mRNA expression is coordinated with promoter H3K9me2 occupation. **(A)** Genes with increased H3K9me2 occupation on their promoters in SCZ samples demonstrate decreased mRNA expression. *GPHN, CDHR2, FURIN, GPX6, OXT*, and *NRGN* all demonstrated significantly increased promoter occupancy of H3K9me2. After the ChIP-seq discovery phase in the initial 15 NPC and 15 SCZ brain samples, an additional 17 NPC, 7 individuals with SCZ, and 19 individuals with BPD samples were included and processed for mRNA expression of these genes, all but *NRGN* showed decreased expression in SCZ when compared to NPC or individuals with BPD samples. **p < 0.01, ***p < 0.005. A table of statistical analysis is provided in Supplementary Table 1. **(B)** Genes with decreased H3K9me2 occupation on their promoters in SCZ samples demonstrate increased mRNA expression. *AKT3, C4B, CDH20*, and *LIPJ* were four genes found to have decreased H3K9me2 promoter occupancy in the SCZ brain samples. When processed for mRNA expression, *AKT3*, *C4B*, and *CDH20* showed increased mRNA expression, but *LIPJ* did not.

**FIGURE 2:**
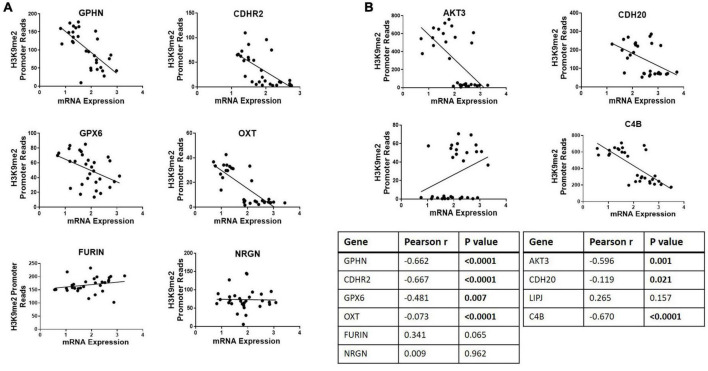
Correlational analysis of H3K9me2 promoter occupation and mRNA expression. Scatterplots showing correlations between H3K9me2 promoter DiffReps reads and mRNA levels of the corresponding gene. **(A)** Promoter loci where H3K9me2 occupancy is significantly increased in SCZ compared to NPC. Promoters GPHN, CDHR2, GPX6, and OXT show H3K9me2 diffReps reads have significant negative correlation with their corresponding mRNA levels. **(B)** Promoter loci where H3K9me2 occupancy is significantly decreased in SCZ compared to NPC. Promoters AKT3, CDH20, and C4B show H3K9me2 diffReps reads have significant negative correlation with their corresponding mRNA levels. Pearson’s r and *P*-values for each loci are shown in the insert table.

Ten genes were selected for qPCR analysis using a larger cohort. Five genes (*GPHN, CDHR2, FURIN, GPX6, and OXT)* of the six genes which showed higher H3K9me2 promoter occupancy also resulted in significantly decreased mRNA expression in individuals with SCZ compared to NPC (Figure 1). Three of the four genes with decreased H3K9me2 promoter occupation in individuals with SCZ (*AKT3*, *C4B*, and *CDH20*) showed significantly increased mRNA expression in individuals with SCZ compared to NPC. When we include the individuals with BPD samples, they exhibit a similar level of mRNA expression to NPC for all 10 genes examined, indicating the effect is specific to individuals with SCZ (Figure 1). Next we performed a correlation analysis between individual promoter H3K9me2 ChIP-Seq reads and mRNA expression of the occupied gene (Figure 2). For all subjects, seven of the 10 genes analyzed were found to have highly significant negative correlations, as shown in Figure 2, supportive of the repressive nature of the H3K9me2 assembly. Of the genes that did not reach significance in their correlations (FURIN, NRGN, and LIPJ), only FURIN achieved significance between individuals with SCZ compared to NPC in the mRNA expression data. We suspect this to be the case due to the closeness in average promoter reads (169.84 vs. 166.46) between the NPC and individuals with SCZ groups. NRGN and LIPJ are perhaps the most curious of all genes analyzed, as the diffReps analysis provided what appeared to be significant enrichment of H3K9me2 on promoters between the NPC and individuals with SCZ groups. Still, neither gene reached significance in either the mRNA expression data or correlational analysis.

### Enrichment of GWAS signals from the PGC3 schizophrenia analysis

To match our list of heterochromatinized gene promoters to the most recent PGC3 SCZ GWAS findings we filtered at the FDR < 0.05 level. We matched 143 promoters that are differentially occupied with H3K9me2 to the significantly associated gene identities form the PGC3 SCZ study (32). We identified 19 promoters (5 with increased heterochromatin and 14 with reduced heterochromatin) which also had significant (*P* < 5e-08) SCZ GWAS signals (Supplementary Table 2). This PGC3 enrichment coordinated with promoter heterochromatin levels was significant (hypergeometric distribution test *p* = 0.022).

### Potential confounding variables

SPSS was used to analyze the possible effects of several confounding factors on the diffReps and qPCR data. There was no significant difference in either diffReps or qPCR data when we compared the individuals with SCZ group with and without antipsychotics, the individuals with BPD group with and without antipsychotics, individuals with SCZ and individuals with BPD grouped together and compared with and without antipsychotics. We found no significant effect of demographic variables; age, sex, brain pH, PMI among the three groups (NPC, SCZ, and BPD), indicating that these variables will not affect our conclusions.

### Epigenetic enzymes/proteins underwriting heterochromatin assembly

**FIGURE 3:**
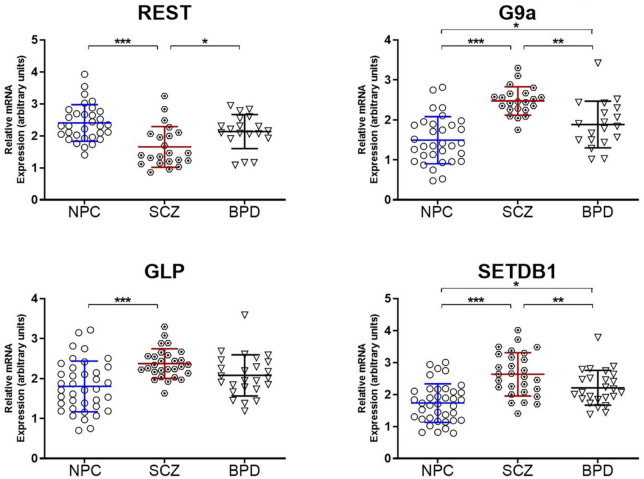
Epigenetic enzymes underwriting heterochromatin formation. Relative mRNA expression levels in the PFC of Repressor element-1 silencing transcription factor (REST) and H3K9 histone methyltransferases (G9a, GLP, and SETDB1). Data is shown as fold change (FC) ± S.E.M. (error bars) after normalization to GAPDH and case compared to NPC. NPC (*n* = 32), individuals with SCZ (*n* = 22), individuals with BPD (*n* = 19). *p ≤ 0.05, **p ≤ 0.01, ***p ≤ 0.001.

Because the target molecule in this study was the assembly of heterochromatin, we also measured mRNA levels of several canonical enzymes critical in producing heterochromatin. These include three HMTs responsible for the H3K9 methylation; GLP and G9A primary for H3K9me2 and SETDB1 for H3K9me3. In addition, we also measured the REST protein (RE1-Silencing Transcription factor), an adaptor/platform protein coordinating heterochromatin assembly alongside other repressor proteins (33, 34). Binding of REST protein to its cognate motif results in an accumulation of H3K9me2/3 heterochromatin (34). The results are presented in Figure 3, indicating an increase in the three measured methyltransferases (GLP, G9A, and SETDB1) and a decrease in the REST protein. Together these results could suggest a chromatin environment conducive to heterochromatin formation in the brain of individuals diagnosed with SCZ.

## Discussion

**FIGURE 4:**
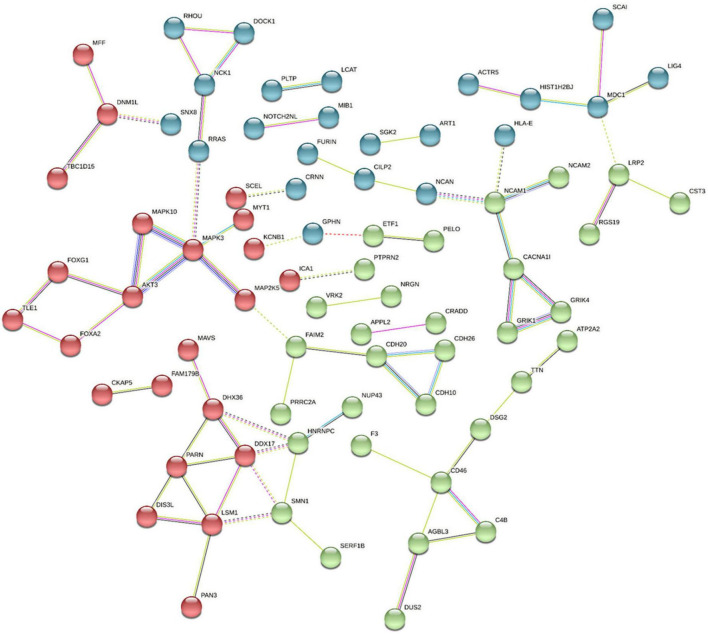
Pathway and Gene Ontology analysis. Using the Cytoscape STRING app, a network was retrieved for the 159 promoters with differentially modified H3K9me2 occupancy. Genes with significantly increased or decreased H3K9me2 levels were selected by the diffReps program using a *p*-value > 0.05 and were entered in the “Network and Enrichment Analysis” within the STRING app. With a confidence cutoff of 0.4, kmeans clustering was used to generate 3 clusters (red, blue, and green). Resulting associations within and between clusters is depicted with solid and dotted lines, respectively. Different color lines indicates different type of interactions. (Cyan-from curated databases; Magenta-experimentally determined; Blue-gene co-occurrence; green-from text mining; Black-coexpression; lilac-protein homology; Red gene fusions).

We have demonstrated that genome-wide sequencing of H3K9me2-bound regions of DNA can provide valuable insight into differentially modified regions and inform correlated mRNA expression levels in the postmortem frontal cortex tissue of individuals with schizophrenia and control subjects. As this was the first sequencing study of its nature for this modification, some gene selections were based on previous disease associations, but also some novel exploratory genes were selected based on their statistical significance and fold change. Using the stringApp in Cytoscape we retrieved functional enrichment analysis results for the 159 H3K9me2 differentially occupied promoter sites listed in Supplementary Table 1 (Figure 4). To visualize and identify groups of proteins that exhibit similar changes in H3K9me2 occupancy we used the K-means clustering method in STRING. This resulted in a network with three clusters, an average local clustering coefficient of 0.36, and an average node degree of 1.04. Identifying enriched genes involved mainly in neurotransmitter/receptor neuronal function, hormone/hormone receptor function, and the immune system. Additionally, we see a high degree of connectivity of AKT3-MAPK3-MAPK10 protein network. All analyses were performed using Cytoscape version 3.9.1, stringApp version 1.7.0, and clusterMaker2 version 2.2. Connections with the Kinase signaling network noted is exciting given the role of MAPK pathways in modifying heterochromatin (35). The nuclear kinase mitogen- and stress-activated protein kinase 1 regulates hippocampal chromatin remodeling in memory formation. Furthermore, our lab has previously demonstrated the role of kinase signaling in “disassembling” heterochromatin using the antipsychotic risperidone (36).

H3K9me2 is known to occupy gene-rich territories and is known to be modifiable using pharmacology directed to one of its primary methylating enzymes G9a. Heterochromatin has durability and is likely responsible for the reduced expression of non-neuronal genes as in the heterochromatin assemblies coordinated with the REST/Co-REST adaptor proteins (37).

### Heterochromatin regulation of synaptic genes

Gephyrin (*GPHN*) is a scaffold protein that docks GABAa receptors to the postsynaptic membrane (38). Abnormal GPHN clustering during neurodevelopment is associated with the pathogenesis of neurological, neurodevelopmental, and psychiatric disorders (39), with exonic deletions implicated in the risk for schizophrenia, autism, and epilepsy development (40, 41). GPHN SNPs have also been associated with schizophrenia (41). Furthermore, a recent study showed that enrichment of copy-number variations in GABAergic genes from individuals with schizophrenia that were identified duplications and deletions in the region of GPHN (42). Deficient GPHN function, as indicated by decreased expression in our samples, impairs inhibitory GABAergic synaptic activity, which results in hyper-excitability of neural cells, as seen in epilepsy (43).

Cadherin family members *CDHR2* and *CDH20* are intercellular adhesion molecules associated with psychiatric disorders (44–46). Variable expression of these proteins may alter synaptic connectivity and information processing in the developing brain. Epilepsy, autism, bipolar, and schizophrenia are associated with cadherin dysfunction, mostly in genome-wide association studies (44, 46). Our expression analysis revealed increased H3K9me2 and decreased expression of CDHR2; decreased H3K9me2, and increased expression of CDH20, suggesting that symptoms typically reported by individuals with schizophrenia may be caused by dysregulation spread throughout the cadherin system.

Neurogranin (*NRGN*) is a neuron-specific calmodulin binding protein, expressed in postsynaptic dendritic spines modulating the activity of downstream calmodulin-Ca^2+^-dependent enzymes that function in the neuroplasticity mechanisms of learning and memory (47). Altered mRNA expression is demonstrated in postmortem brain tissue of individuals with schizophrenia (48–50). Altered CSF levels of NRGN are associated with the impairments in cognition seen in both Alzheimer’s and Parkinson’s disease (51).

### Heterochromatin regulation of catalytic activity

*FURIN* is necessary for axonal growth and development of dendritic arbors, a process linked to neurological diseases of the CNS (52). Knockdown of *FURIN* in human neural progenitor cells results in abnormal neuronal migration (53). In a study examining RNA-seq from over 500 dorsolateral prefrontal cortices, FURIN emerged as one of five single-gene loci implicated in schizophrenia. Decreased expression of this gene due to heterochromatinization could result in disruption of many key systems, potentially contributing to comorbidities associated with schizophrenia.

Glutathione Peroxidase 6 (*GPX6*) is part of a family of enzymes that protect cells from oxidative stress, resulting from an imbalance between reactive oxygen species (ROS) production and deficiency of antioxidants to process the ROS, leading to deleterious peroxidations of lipids, proteins, and DNAs. The brain is especially vulnerable to oxidative damage due to its high utilization of oxygen, high content of oxidizable polyunsaturated fatty acids, and the presence of redox-active metals (54). Oxidative stress is hypothesized to contribute to symptomology reported by individuals with schizophrenia, specifically along the Tryptophan/Kynureine/Quinolinic acid pathway, which can be induced by cytokines such as IFNγ, long noted to be abnormally regulated in schizophrenia (55). Developmental dysregulation of glutathione synthesis combined with environmental risk factors that generate oxidative stress may result in deficits in neural connectivity and synchronization observed in individuals with schizophrenia (56–58).

Though Lipase family member J (*LIPJ*) is not well studied, the general lipase family increases after antipsychotic treatment (59). Therefore, our non-significant mRNA expression results may be a result of the variable antipsychotic dosing present in our sample. We have also shown that antipsychotic treatment *in vitro* can decrease heterochromatin levels and increase promoter phosphorylation and mRNA expression of genes that are tolerized (heterochromatinized) *via* an endotoxin tolerance paradigm, while other promoters do not respond in this way (36, 60). This targeted response to antipsychotic treatment could be an explanation for the LIPJ and NRGN genes that did not show coordination with their H3K9me2 promoter occupation levels.

The AKT Serine/Threonine Kinase 3 (*AKT3*) genomic locus is a replicated GWAS signal in individuals with schizophrenia [Schizophrenia Working Group (61)]. AKT signaling plays a critical role in cell growth, proliferation, survival, differentiation, and metabolism. AKT3 is highly expressed in the brain, with expression peaking during development, indicating a crucial role in cognitive function (62). AKT3 genetic abnormalities result in intellectual disabilities (63–67), and deficits in temporal order discrimination and spatial memory are seen in AKT3 knockout mice (68). Protein levels of AKT3 family member AKT1 are significantly reduced in lymphocytes and the brain of individuals diagnosed with schizophrenia (69). However, our findings seem to conflict, as AKT3 expression is increased in postmortem prefrontal cortices in individuals diagnosed with schizophrenia (Figure 1).

Oxytocin is a peptide hormone acting on both central and peripheral targets to decrease cortisol release in response to social stresses (70). In individuals with schizophrenia, plasma oxytocin levels are elevated in response to trust-related interactions, an effect not seen in normal controls (71). Low oxytocin levels also correlate with negative symptoms in individuals with schizophrenia (72). Our data found significant H3K9me2 enrichment on the *OXT* promoter, correlating with significantly decreased oxytocin mRNA levels in postmortem brain tissue of individuals with schizophrenia. Due to the function of the hormone, it is hypothetically valid to suggest that heterochromatin promoter occupancy and decreased oxytocin mRNA expression could limit the plastic response to social stress or interactions seen in individuals with schizophrenia.

The C4 locus on chromosome 6 has a strong association with postmortem brain tissue in individuals with schizophrenia (31, 73) and expresses two isotype molecules, C4A and C4B (74). As both complements serve a crucial role in synaptic pruning (75), it follows that dysregulation of this process can result in aberrant connections that can underlie neurological disorders. Most recently, our lab showed that C4A mRNA expression is positively associated with psychotic symptomology, including the severity of delusions (76). Our data support a linkage between aberrations in postmortem brain tissue in individuals with schizophrenia, as we found that at the C4B promoter, H3K9me2 was significantly decreased, resulting in increased mRNA levels.

### Epigenetic enzymes/proteins underwriting heterochromatin assembly

We have previously demonstrated elevated levels of both mRNA and protein in two separate tissues obtained from individuals with schizophrenia; parietal cortical samples from the Stanley Foundation Neuropathology Consortium and lymphocyte samples from the University of Illinois at Chicago (UIC) (14). In both tissues types, we measured mRNA expression of HMTs, GLP, G9a, and SETDB1 *via* real-time RT-PCR and H3K9me2 levels *via* western blot. We have replicated this in our current study using frontal cortex from the Harvard Brain Tissue Resource Center. Elevation of the mRNA of these HMTs suggest a cellular/neuronal environment conducive to heterochromatin formation. We additionally measured mRNA levels of REST protein, which is a platform adaptor protein that coordinates the multicomponent assembly of Heterochromatin proteins, and note that expression levels are differentially reduced in individuals with schizophrenia.

### Limitations

There are several sources of variation that could misalign our results when comparing DNA promoter occupancy (estimation of DNA sequences from the smaller ChIP-Seq subset) to its downstream gene expression (mRNA measured by qRT-PCR and presented in Figure 1). These could include variations in the expanded cohort utilized purely for qRT-PCR, unidentified sources of gene regulation that could impact gene mRNA expression levels, and additional regulatory factors acting downstream of the promoter. For example, the NRGN locus shows significantly increased H3K9me2 occupancy (refer to *p*-value in Table 2), while the LIPJ locus has significantly decreased H3K9me2 occupancy (refer to *p*-value in Table 2). While conceptually, we would see the coordination of promoter occupancy and mRNA expression, these two measurements (ChIP-seq vs. qRT-PCR) are uncoupled for these two loci.

We did not perform ChIP-seq on subjects with BPD, and therefore, we can only state that heterochromatin itself is differentially located, in SCZ, on the promoter sites provided in Supplementary Table 1 and note that the mRNA expression of these promoters in BPD is not different from the mRNA expression in NPC, allowing us to conclude that the selected promoters are “heterochromatinized” in SCZ and not BPD samples.

There are universal confounds in all translational or clinical tissue-oriented studies including the effect of pharmacology/psychotropics, obesity, drugs of abuse, trauma, or environmental factors related to psychosocial disparities and these have not been resolved. Limitations of ChIP-Seq directed to a specific histone modification are impacted by the heterogeneous population of neuronal/non-neuronal cells. This is approached with techniques such as laser capture dissection of unique neuronal populations or FACS sorting according to neuronal markers (77), but this was not feasible given the tissue requirements of ChIP-Seq.

## Conclusion and summary

We have demonstrated the experimental identification of specific gene promoters differentially occupied by H3K9me2 heterochromatin in the PFC of postmortem brain tissue in individuals diagnosed with schizophrenia. We have also demonstrated a functional effect of promoter occupation by this modification on mRNA gene expression in a coordinated manner. We demonstrate differential modification levels on the promoters of several genes that have previously been linked to individuals with schizophrenia, in addition to genes that are novel to the disorder. Some of the genes featured here offer coherent functional explanations to established hypotheses (i.e., Gephyrin expression and the hypothesis of excitatory and inhibitory synapse imbalance as it relates to individuals with schizophrenia), while others will require further exploration to determine their role in the development or maintenance of disease.

Our ability to demonstrate differences in heterochromatin along specific gene promoters in the brains of individuals diagnosed with schizophrenia opens up new hypotheses and therapeutic targets. We have earlier noted that heterochromatin assemblies are durable and can persist for the life span especially in neurons (78). Theoretically, they possibly serve as repositories for the accumulation of psychological, metabolic or pharmacological inputs over extended periods (79). In treatment resistant individuals, the blockade of gene promoters by durable and stable repressive heterochromatin could explain why targeting of membrane monoamine synaptic receptors is not adequate in disassembling these deep genomic structures and the inability to activate the underlying gene networks (79). Our findings suggest that alternate/parallel signaling pathways capable of targeting genomic heterochromatin may provide a supplemental advantage.

Considering this approach, we have elsewhere demonstrated that signaling along the kinase pathways in cell cultures can target heterochromatin assemblies by regulating histone phosphorylation (36). Because heterochromatin is a stable modification and in equilibrium with its regulatory factors, its measurement along specific gene networks in the neuron would allow a reverse engineering approach to study “what stress” deposits heterochromatin on “which network.” This approach has been utilized using defined immunological stimuli to assemble heterochromatin on pre-defined immune gene promoters (15, 36, 80–82).

To our knowledge, this is the first study of H3K9me2 based heterochromatin promoter assembly in the postmortem brain tissue in individuals diagnosed with schizophrenia and is directly relevant to gene expression. These modifications may be particularly relevant in individuals who are resistant to available psychotropics, suggesting gene networks that are not receptive to usual monoamine receptor signaling. Future studies aiming to reveal new potential targets for treatment advancement will attempt to map the distribution of other restrictive chromatin assemblies such as those originating with H3K27me3, as well as mapping regions of the genome that are primed for transcription such as those occupied by H3K4me1/2/3.

## Data availability statement

The original contributions presented in the study are publicly available. The data presented in the study are deposited in the GEO repository, accession number GSE215991. This data can be found here: https://www.ncbi.nlm.nih.gov/geo/query/acc.cgi?acc=GSE215991.

## Ethics statement

The studies involving human brain samples were reviewed and approved by the University of Illinois Institutional Review Board. Brain samples utilized for this study were obtained from the Harvard Brain Tissue Resource Center.

## Author contributions

RS was PI for this grant, coordinated the interpretation, data analysis, manuscript preparation and submission. CL and CX helped with data analysis and wrote part of the methods for the manuscript. HR, KC, CR, and HG provided critical scientific input on data analysis and manuscript preparation. AG provided aliquots of brain samples from the Harvard Brain Tissue Resource Center. All authors provided critical edits to this manuscript.

## Footnotes

1
https://www.med.unc.edu/pgc/download-results
