# Supplementary material for: Differential H3K9me2 heterochromatin levels and concordant mRNA expression in postmortem brain tissue of individuals with schizophrenia, bipolar, and controls
Source: Front Psychiatry. 2022 Oct 26;13:1006109. doi: 10.3389/fpsyt.2022.1006109 (PMC9644155; doi:10.3389/fpsyt.2022.1006109)
Supplement: Supplementary file 1 [file Data_Sheet_1.PDF]

Supplementary Figures and Tables

Supplementary Figure 1. Quality control of ChIP, sequencing, and read alignment

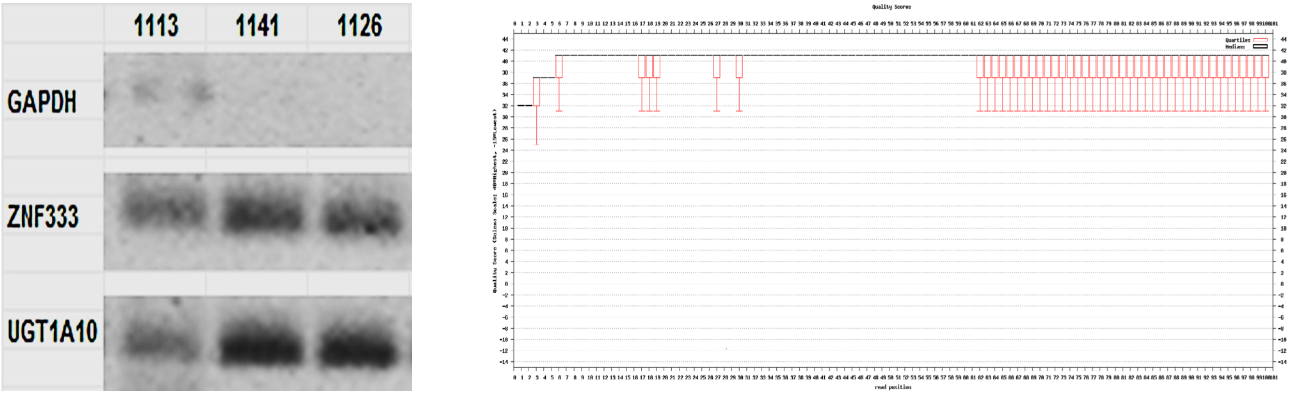

(A). Quality control for the H3K9me2 antibody. Representative gel images from qPCR of immunoprecipitated DNA from three different samples (sample ID on top row) for GAPDH promoter (negative control, top), ZNF333 promoter (positive control, middle), and UGT1A10 promoter (positive control, bottom). The sample 1113 would not be considered as a successful experiment and thus would not be used for library preparation or sequencing. (**B).** FASTQC quality results showing the average sequence quality of all of the reads in a data file. The base position in the read is displayed along the x-axis, while the phred score is along the y-axis. A phred score is a probability calculation that the sequencer has determined the correct base. Phred scores above 30 (green) indicate excellent quality and base confidence of 99.99% or higher.

3

Supplementary Figure 2. Read alignment visualization using UCSC Genome Browser

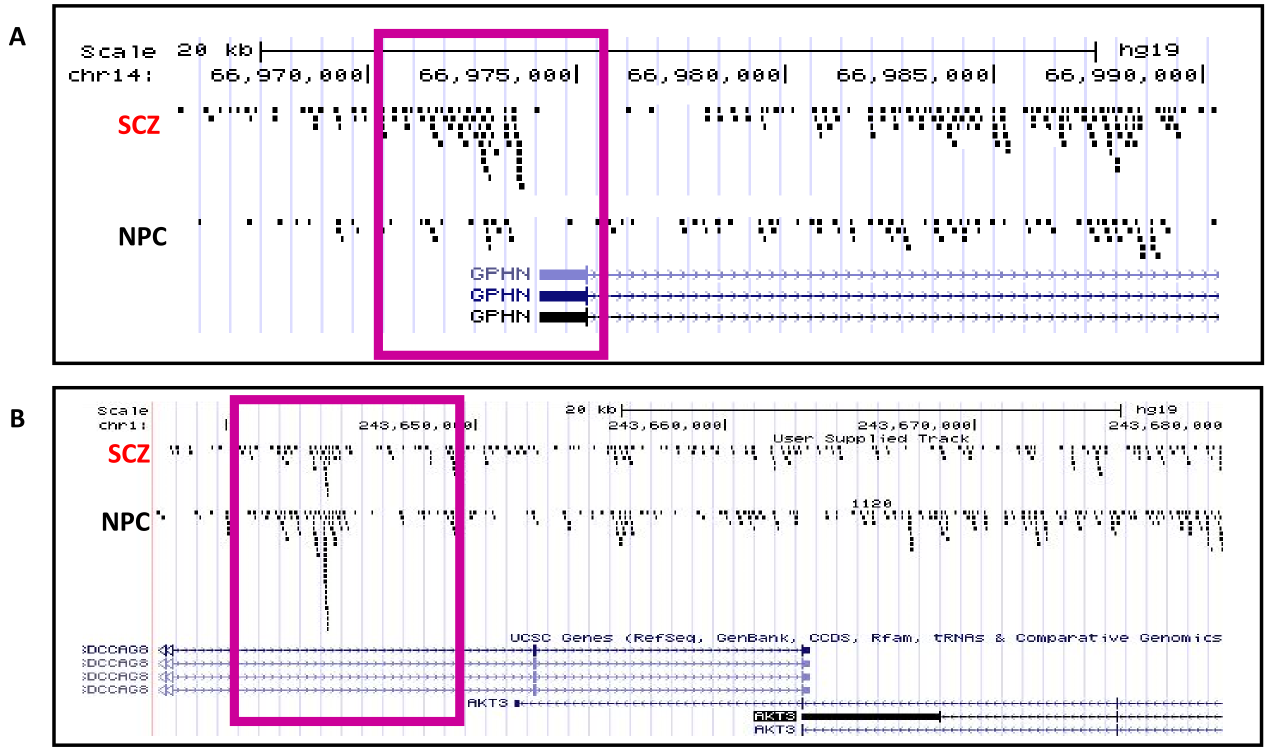

Read alignment visualized with UCSC Genome Browser. Each short read was matched to its position on the genome using Bowtie2. These aligned files can then be visualized by a number of programs; custom track creation in UCSC Genome Browser is demonstrated here. When the browser is localized to each validated promoter (GPHN, FURIN, CDHR2, GPX6, NRGN, OXT, AKT3, C4B, CDH20, LIPJ). The location and distribution of aligned reads for two of the ten genes analyzed showing **(A)** increased (GPHN) H3K9me2 promoter occupancy and **(B)** decreased (AKT3) H3K9me2 promoter occupancy in the SCZ compared to NPC sample. This is a visual comparison from selected samples as a 'proof of principle' illustration of the location and extent of the H3K9me2 modification. This figure is not meant for statistical inference.

Supplementary Table 1. Gene set of all 159 differentially modified promoters

Gene name	SZ avg read n=15	NPC avg reads n=15	H3K9me2 occupancy	log2FC	P-value	P-adj
**FURIN**	169.84	166.46	Up	0.03	1.08E-10	8.57E-07
RRAS	71.88	52.49	Up	0.45	0.000309899	0.098449394
**NRGN**	86.06	62.46	Up	0.46	0.000260971	0.091196263
TLE1	113.65	79.65	Up	0.51	0.000327037	0.101585095
VRK2	285.71	185.51	Up	0.62	0.000408156	0.108426843
NCK1	26.19	16.84	Up	0.64	0.000254744	0.091196263
RAI1	329.42	208.26	Up	0.66	0.000287013	0.095520771
NCAN	261.6	164.16	Up	0.67	0.000224947	0.083743424
PLCL1	224.62	141.6	Up	0.67	0.000276772	0.09435897
MIR4529	51.81	31.72	Up	0.71	9.44E-05	0.045514749
RENBP	52.15	31.96	Up	0.71	0.000300981	0.097839712
HIST1H2BJ	61.12	36.98	Up	0.73	8.40E-06	0.008385843
MIR548AJ2	30	18.05	Up	0.73	0.000147247	0.062370247
SMN1	409.25	237.8	Up	0.78	1.20E-11	9.88E-09
SERF1B	154.92	76.69	Up	1.01	2.08E-07	9.43E-05
ICA1	294.35	139.43	Up	1.08	5.42E-14	5.15E-11
**GPX6**	68.9	32.26	Up	1.09	7.51E-07	0.000954394
FLJ36000	231.09	96.82	Up	1.25	5.28E-14	5.03E-11
USP17	172.99	71.26	Up	1.28	3.85E-11	3.07E-08
**GPHN**	140.72	54.22	Up	1.38	2.89E-10	2.13E-07
SNAR-C2	121.35	45.7	Up	1.41	2.50E-09	1.66E-06
HNRNPC	117.48	41.05	Up	1.52	5.69E-10	4.07E-07
AHRR	125.23	42.6	Up	1.56	7.06E-11	5.52E-08
MRPS14	108.44	36.41	Up	1.57	9.27E-10	6.47E-07
ART1	112.32	35.63	Up	1.66	1.04E-10	7.94E-08
FAM27A	152.34	46.48	Up	1.71	1.29E-14	1.28E-11
LOC284412	122.64	34.08	Up	1.85	3.14E-13	2.87E-10
LOC728716	60.68	16.27	Up	1.9	1.75E-07	8.06E-05
MUC2	77.46	19.36	Up	2	1.00E-09	6.96E-07
TBC1D15	72.3	17.82	Up	2.02	2.81E-09	1.85E-06
FAIM2	82.62	20.14	Up	2.04	1.71E-10	1.28E-07
LOC642236	101.99	24.01	Up	2.09	5.73E-13	5.16E-10
LOC100132352	103.28	24.01	Up	2.1	3.04E-13	2.79E-10
SNAR-A3	81.33	17.82	Up	2.19	3.17E-11	2.55E-08
BAGE	121.35	25.56	Up	2.25	2.22E-16	2.46E-13
XPR1	121.35	25.56	Up	2.25	2.22E-16	2.46E-13
LOC728373	85.21	17.82	Up	2.26	4.41E-12	3.71E-09
CRADD	72.3	14.72	Up	2.3	1.16E-10	8.87E-08
AGBL3	73.59	14.72	Up	2.32	5.93E-11	4.66E-08
IDE	65.84	13.17	Up	2.32	5.96E-10	4.25E-07
KIAA0825	65.84	13.17	Up	2.32	5.96E-10	4.25E-07
MIB1	46.48	9.3	Up	2.32	1.97E-07	9.03E-05
DOCK1	68.42	13.17	Up	2.38	1.53E-10	1.16E-07
PELI2	59.39	10.84	Up	2.45	1.23E-09	8.46E-07
APPL2	65.84	11.62	Up	2.5	9.63E-11	7.41E-08
DNER	63.26	10.84	Up	2.54	1.50E-10	1.13E-07
MAVS	41.31	6.97	Up	2.57	1.96E-07	9.00E-05
MDC1	41.31	6.97	Up	2.57	1.96E-07	9.00E-05
MAP2K5	56.8	9.3	Up	2.61	7.27E-10	5.15E-07
**CDHR2**	61.97	10.07	Up	2.62	1.13E-10	8.67E-08
SCAI	52.93	8.52	Up	2.64	2.28E-09	1.52E-06
LOC728369	69.71	10.84	Up	2.68	4.21E-12	3.55E-09
GRIK4	55.51	8.52	Up	2.7	5.35E-10	3.84E-07
SNX8	37.44	5.42	Up	2.79	2.18E-07	9.67E-05
SPINT4	37.44	5.42	Up	2.79	2.18E-07	9.67E-05
SRBD1	37.44	5.42	Up	2.79	2.18E-07	9.67E-05
TERC	37.44	5.42	Up	2.79	2.18E-07	9.67E-05
SIRPD	49.06	6.97	Up	2.81	2.45E-09	1.63E-06
NOTCH2NL	50.35	6.97	Up	2.85	1.16E-09	8.04E-07
PDXDC1	50.35	6.97	Up	2.85	1.16E-09	8.04E-07
ZNF233	58.09	7.75	Up	2.91	4.07E-11	3.25E-08
SCEL	45.18	5.42	Up	3.06	2.31E-09	1.54E-06
ALCAM	52.93	6.2	Up	3.09	7.86E-11	6.10E-08
NCAM1	46.48	5.42	Up	3.1	1.07E-09	7.41E-07
NCAM2	46.48	5.42	Up	3.1	1.07E-09	7.41E-07
NCK1	33.57	3.87	Up	3.12	2.04E-07	9.23E-05
NUP43	33.57	3.87	Up	3.12	2.04E-07	9.23E-05
OR2B3	33.57	3.87	Up	3.12	2.04E-07	9.23E-05
**OXT**	33.57	3.87	Up	3.12	2.04E-07	9.23E-05
PAN3	33.57	3.87	Up	3.12	2.04E-07	9.23E-05
PARN	33.57	3.87	Up	3.12	2.04E-07	9.23E-05
PELO	33.57	3.87	Up	3.12	2.04E-07	9.23E-05
PLTP	33.57	3.87	Up	3.12	2.04E-07	9.23E-05
PRRC2A	33.57	3.87	Up	3.12	2.04E-07	9.23E-05
LCORL	45.18	4.65	Up	3.28	6.44E-10	4.59E-07
PHACTR3	41.31	3.87	Up	3.42	1.78E-09	1.21E-06
EFCAB1	45.18	3.87	Up	3.54	1.59E-10	1.20E-07
PRIM2	38.73	3.1	Up	3.64	2.08E-09	1.40E-06
PRIMA1	38.73	3.1	Up	3.64	2.08E-09	1.40E-06
FAM179B	42.6	3.1	Up	3.78	1.78E-10	1.33E-07
PTPRN2	36.15	2.32	Up	3.96	2.20E-09	1.47E-06
MAPK10	34.86	1.55	Up	4.49	8.44E-10	5.92E-07
ZNF571	1.29	56.55	Down	-5.45	2.22E-16	2.46E-13
**LIPJ**	1.29	53.45	Down	-5.37	1.55E-15	1.62E-12
ACTR5	141.46	3379.84	Down	-4.58	6.17E-07	0.000954394
**AKT3**	26.74	515.22	Down	-4.27	1.38E-07	0.000384508
ATP2A2	58.25	1127.07	Down	-4.27	2.78E-07	0.000485405
BAG5	24.47	317.2	Down	-3.7	2.63E-07	0.000485405
ZNF570	10.33	85.98	Down	-3.06	2.22E-16	2.46E-13
LOC100507244	5.16	37.18	Down	-2.85	1.74E-07	8.02E-05
LOC653486	5.16	37.18	Down	-2.85	1.74E-07	8.02E-05
DNM1L	14.2	94.5	Down	-2.73	4.44E-16	4.81E-13
BRP44	32.22	209.52	Down	-2.7	7.41E-07	0.000954394
DIS3L	15.49	97.6	Down	-2.66	4.44E-16	4.81E-13
ELF2	16.78	99.92	Down	-2.57	5.55E-16	5.96E-13
TTC34	19.36	109.22	Down	-2.5	1.11E-16	1.25E-13
ZNF568	24.53	119.29	Down	-2.28	2.22E-16	2.46E-13
FERMT1	25.82	119.29	Down	-2.21	6.66E-16	7.11E-13
DEFB115	28.4	125.48	Down	-2.14	4.44E-16	4.81E-13
ZNF569	11.62	51.12	Down	-2.14	2.20E-07	9.67E-05
LOC100132077	12.91	54.22	Down	-2.07	1.71E-07	7.88E-05
KLK13	32.27	130.91	Down	-2.02	1.33E-15	1.40E-12
ZNF431	38.73	147.95	Down	-1.93	2.22E-16	2.46E-13
LOC100131208	36.15	137.88	Down	-1.93	1.67E-15	1.73E-12
FADS2	38.73	144.85	Down	-1.9	6.66E-16	7.11E-13
CST3	41.31	151.05	Down	-1.87	3.33E-16	3.65E-13
ZNF19	16.78	61.19	Down	-1.87	2.17E-07	9.67E-05
ZNF429	43.89	156.47	Down	-1.83	2.22E-16	2.46E-13
DDX17	43.89	154.92	Down	-1.82	4.44E-16	4.81E-13
ZNF345	47.77	163.44	Down	-1.77	2.22E-16	2.46E-13
DPH1	47.77	161.89	Down	-1.76	5.55E-16	5.96E-13
DSG2	47.77	161.89	Down	-1.76	5.55E-16	5.96E-13
LGR6	46.48	157.24	Down	-1.76	1.55E-15	1.62E-12
LIG4	46.48	157.24	Down	-1.76	1.55E-15	1.62E-12
GRSF1	51.64	167.31	Down	-1.7	1.11E-15	1.17E-12
GRIK1	52.93	169.64	Down	-1.68	1.11E-15	1.17E-12
TTN	23.24	72.81	Down	-1.65	2.18E-07	9.67E-05
USP15	23.24	72.81	Down	-1.65	2.18E-07	9.67E-05
FOXG1	56.8	176.61	Down	-1.64	8.88E-16	9.40E-13
GPR155	56.8	176.61	Down	-1.64	8.88E-16	9.40E-13
ZDHHC17	63.26	191.33	Down	-1.6	2.22E-16	2.46E-13
ZNF10	63.26	192.1	Down	-1.6	2.22E-16	2.46E-13
CRNN	61.97	188.23	Down	-1.6	3.33E-16	3.65E-13
F3	61.97	186.68	Down	-1.59	6.66E-16	7.11E-13
NANP	25.82	77.46	Down	-1.58	2.01E-07	9.20E-05
CTSA	67.13	195.97	Down	-1.55	4.44E-16	4.81E-13
CDH26	68.42	199.07	Down	-1.54	3.33E-16	3.65E-13
HLA-E	67.13	193.65	Down	-1.53	1.22E-15	1.28E-12
FOXA2	68.42	196.75	Down	-1.52	8.88E-16	9.40E-13
**CDH20**	74.88	209.92	Down	-1.49	3.33E-16	3.65E-13
SGK2	30.98	85.98	Down	-1.47	2.13E-07	9.65E-05
MFF	32.27	88.3	Down	-1.45	1.97E-07	9.01E-05
LPIN3	33.57	90.63	Down	-1.43	1.81E-07	8.31E-05
KIAA1671	80.04	214.56	Down	-1.42	1.33E-15	1.40E-12
RHOU	36.15	94.5	Down	-1.39	2.03E-07	9.23E-05
CST8	90.37	234.7	Down	-1.38	4.44E-16	4.81E-13
LRFN3	37.44	96.82	Down	-1.37	1.84E-07	8.43E-05
MYT1	40.02	100.7	Down	-1.33	1.99E-07	9.10E-05
FGL1	107.15	258.72	Down	-1.27	8.88E-16	9.40E-13
**C4B**	256.53	619.55	Down	-1.27	0.006282752	0.286791739
LRP2	46.48	110.77	Down	-1.25	1.96E-07	8.99E-05
CST7	116.19	274.98	Down	-1.24	4.44E-16	4.81E-13
RGS19	51.64	118.51	Down	-1.2	2.03E-07	9.23E-05
ROMO1	65.84	139.43	Down	-1.08	2.06E-07	9.33E-05
CACNA1I	32.35	65.41	Down	-1.02	8.37E-07	0.000975465
CD46	191.75	383.2	Down	-1	0.002585163	0.238652273
CILP2	18.84	34.06	Down	-0.85	1.94E-07	0.000451249
CKAP5	17.6	30.2	Down	-0.78	9.08E-06	0.008459651
DUS2L	36.8	61.97	Down	-0.75	0.001938237	0.222071144
EPC2	495.09	834.45	Down	-0.75	0.008293238	0.315866163
FAUP2	129.24	213.11	Down	-0.72	0.003768947	0.256492453
ETF1	34.25	56.52	Down	-0.72	0.013851614	0.426471065
MAPK3	29.1	46.33	Down	-0.67	3.49E-05	0.023219442
LSM1	18.06	28.07	Down	-0.64	3.05E-05	0.021324124
LCAT	38.58	59.72	Down	-0.63	2.97E-05	0.021324124
MAU2	20.97	31.33	Down	-0.58	4.46E-05	0.02834177
KCNB1	150.63	223.42	Down	-0.57	2.55E-05	0.019777626
SMEK1	387.3	545.32	Down	-0.49	2.14E-07	9.66E-05
CDH10	1161.9	1588.7	Down	-0.45	3.33E-16	3.65E-13

Supplementary Table 2. PGC3 SCZ GWAS enrichment analysis summary

Group	# Sig_GWAS_Gene	# Sig_DEG_Gene	# Background_Gene	# Overlap	Enrichment_P
Up	2010	72	25069	5	0.694438
Down		71		14	0.001347
Up & Down		143		19	0.020213

The significantly differentially modified promoters were searched against GWAS of about 25,069 genes from the latest public SCZ GWAS summary statistics from Psychiatric Genomics Consortium (PGC)1. Hypergeometric distribution test was performed to see whether they are enriched in the PGC3 SCZ GWAS significant associated genes using the software R 4.1.0. The PGC3 SCZ GWAS summary statistics was downloaded from PGC website (https://www.med.unc.edu/pgc/download-results), and the SNPs were annotated to genes using the software ANNOVAR2 based on hg19_avsnp142 database.

Supplementary Table 3. Primer sequences for qPCR expression analysis.

	Human Q-PCR primer sequences			
**Gene name**	**Gene symbol**	**Forward primer**	**Reverse primer**	**Product length**
Gepyhrin	GPHN	GGAGTTCAATGTGAGGAAGAGGA	ACCGTGTGCCATGACAACA	150
cadherin related family member 2	CDHR2	CGAAGAGGCCCAAGTGAACT	GGATACTCTCACCAGCACCT	214
AKT serine/threonine kinase 3	AKT3	TTTCTCCAAGTTGGGGGCTC	ACTGGCATTTTGCCACTGAAA	215
lipase family member J	LIPJ	GGGTTCACTATCTGTTTGCTGG	CGTTTGGGACCTCTCTGCTT	210
oxytocin/neurophysin I	OXT	CTGCTTCGGGCCCAATATCT	AGTTTCAGCGCTGGGAGAAG	230
complement C4B	C4B	GAGTCTCCAGGTGGGGAGAT	GAGAAGACACCGTGGCAGAA	199
glutathione peroxidase 6	GPX6	TTCCTGGTTGGCTTTGCTCA	AGCTGCCAAGCCTCAATAGG	192
furin, paired basic amino acid cleaving enzyme	FURIN	CAGGATGAATCCCAGGTGCT	TTGCTGCTACCACCCATAGC	237
neurogranin	NRGN	GTTTTGGTTTCGGACGACCC	TCACATGCACACACGGAGAT	263
cadherin 20	CDH20	CTTCTCACTGGACAGGCCG	CGGAGACCTTCGGTCCTAAC	293
RE1 silencing transcription factor	REST	AGCGTCCTGTGTTGGAATGT	GGGAGGGTGGTAAAGTTGGG	209
euchromatic histone lysine methyltransferase 2	G9A	ATAGCAAGGAGGAGGACGGT	CCTCGTTGTCAGTGAGGGTG	219
SET domain bifurcated histone lysine methyltransferase 1	SETDB1	GACGGGAGAGGACAAAAGCA	CAACGTGAGCCACCTCAGAT	254
glucagon like peptide 1 receptor	GLP	AGGCGCTGCTTTTGTTTGAG	CAGAGCTCCAACGATGTGGT	282

8
